# Risk attitudes and personality traits of entrepreneurs and venture team members

**DOI:** 10.1073/pnas.1908375116

**Published:** 2019-08-19

**Authors:** Sari Pekkala Kerr, William R. Kerr, Margaret Dalton

**Affiliations:** ^a^Wellesley Centers for Women, Wellesley College, Wellesley, MA 02481;; ^b^National Bureau of Economic Research, Cambridge, MA 02138;; ^c^Entrepreneurial Management Unit, Harvard Business School, Boston, MA 02163

**Keywords:** entrepreneurs, inventors, innovation, personality, risk

## Abstract

Personality distinctions between entrepreneurs, nonfounder CEOs/leaders, and inventor employees have received limited attention, especially in innovative settings where they are working together. We surveyed these groups, along with other employees of innovative firms, at 4 locations of a prominent innovation and coworking center. Entrepreneurs display the greatest tolerance of risk, even in small gambles, as well as the strongest self-efficacy, internal locus of control, and need for achievement. Nonfounder CEOs/leaders typically sit in between entrepreneurs and employees for personality traits. Entrepreneurs, nonfounder CEOs/leaders, and inventor employees all show more innovative personalities than the noninventor employees in the same companies.

## Significance

We quantify personality traits and risk tolerances among entrepreneurs and their venture team members using a unique and in-depth survey of multiple locations of an innovation and coworking center. Our survey captures respondents’ own perceptions of their personality traits and an observational assessment of their risk tolerance via a prize lottery. These detailed profiles allow us to describe similarities and differences between entrepreneurs, nonfounder CEOs/leaders, inventor employees, and noninventor employees. This survey provides a characterization of these different personality profiles in innovative firms and environments that have all roles present and thus controlling for confounding environment factors.

New ventures have many types of individuals playing important and distinct roles. Entrepreneurs are needed to challenge the status quo and build the foundation and energy for a new approach to a customer need, industry structure, or business function. Nonfounder CEOs/leaders help establish the business processes, operational capabilities, and organization to scale emerging ideas to commercial success. Inventor employees generate new technological capabilities that sit behind high-growth companies. Many other employees staff roles from sales/marketing to customer support to administrative assistance.

While each of these individuals—entrepreneurs, nonfounder CEOs/leaders, inventor employees, noninventor employees—play important roles in innovation and economic growth, relatively little is known about their respective personality traits. This limitation is especially true when isolating innovative firms and environments that have all roles present and thus controlling for confounding environment factors. Understanding these differences provides important insight into team formation for new ventures and also better characterizes potential career trajectories (e.g., whether employees in start-ups appear to have personalities and risk tolerances that might raise their likelihood to become future founders).

We quantify these personality differences using a unique survey of the inhabitants of CIC. CIC was founded in its present format in 2001 as the Cambridge Innovation Center (later formally adopting its acronym as its official business name as it expanded beyond Cambridge, MA). The first and largest CIC facility at One Broadway in Cambridge, MA, is adjacent to and in a building owned by the Massachusetts Institute of Technology (MIT). CIC houses startups, single individuals in coworking spaces, not-for-profit organizations, law firms, venture investors, and satellite offices and R&D laboratories for large corporations.

CIC is, today, the center of Boston’s entrepreneurial and innovation ecosystem. CIC pitches itself as having “More startups than anywhere else on the planet,” and well-known start-up ventures from CIC include Android (later acquired by Google), Carbonite, and Hubspot. In total, CIC-based start-ups have raised over $7 billion in venture capital funding and produced thousands of patents since CIC’s founding in 2001. This level of venture investment into CIC-housed companies exceeds most US states and many nations.

CIC also is home to the innovation laboratories and satellite offices of many large companies. These have included Amazon, Apple, Bayer, Google, and Shell, and legend has it that Apple’s Siri was partly developed at CIC. According to CIC’s data, the industry mix of its firms is about 32% technology, 21% science, 18% business, 13% nonprofit, and 17% other. The scale and diversity of CIC’s tenants generates a unique platform to study entrepreneurs, nonfounder leaders, inventors, and employees working in innovative enterprises.

CIC pioneered the coworking space model, which has risen to popularity with the “sharing economy” (1). CIC offers its clients month-to-month rentals and office management services, which include access to regular and 3D printing, hardware tool shops, conference rooms, information technology and communications infrastructure, and fully stocked communal kitchens. CIC also creates formal and informal networking opportunities, lectures on topics related to startups and innovation, recreational classes like yoga, and proximity to funders, law firms, and other service providers. The weekly Venture Café “happy hour” regularly draws hundreds of participants from the local area and is held at CIC.

CIC has expanded substantially since 2001. At the One Broadway location, CIC grew from 1 floor to 7. CIC expanded to St. Louis in 2014, and it also opened 2 new locations in the Boston area by 2017. It has recently opened facilities in Miami, Philadelphia, Providence, Rotterdam, and Warsaw, as well as a fourth Boston facility. Rapidly scaling, CIC plans to reach 50 global cities by 2026 (2).

The survey of CIC clients was conducted at 3 locations in Boston/Cambridge and the St. Louis facility in 2017. The survey was sent to 5,645 individuals by CIC as part of their annual client survey. A total of 1,334 people participated in the survey (24% response rate). This strong response rate was due, in part, to CIC emphasizing this survey to its tenants as important to complete.

## Materials and Methods

The CIC client agreement specifies that all clients agree to participate in the CIC annual survey, and that these data may be used in research if the client so permits. CIC collaborated with us to design the 2017 survey instrument, which included an “informed consent” statement at the beginning. Upon responding to the survey, clients agreed to having their data be part of the research study. CIC provided the data to us under a nondisclosure agreement. Wellesley College Institutional Review Board (Committee for the Protection of Human Subjects) reviewed the proposal and deemed it exempt from human subjects protections under Exemption 2 (adults) concerning survey procedures where 1) the subjects cannot be readily identified and 2) disclosure of responses would not cause any risk of criminal/civil liability or other personal damage (e.g., financial, employability, or reputation).

The survey was open for 15 wk. CIC sent 1 reminder email per location and hosted a pizza lunch at one of the Cambridge locations. One of our team was present at the lunch to distribute flyers and answer questions. Additionally, a reminder email for that location was sent the previous day so as to be at the top of clients’ inboxes. CIC decided when to close the survey and how to interact with clients during the survey period.

*SI Appendix*, Table S1 provides descriptive statistics on CIC facilities and the survey overall. About 20% of individuals at CIC are identified as head of a firm (e.g., to whom a bill is sent). Our response rates are similar to or exceed typical surveys of entrepreneurs, ranging between 16% and 24%. For those who start the survey, response rates are high for the majority of the questions. Questions regarding demographics all have response rates of over 80%, while questions regarding personality all have response rates of over 75%. Questions with the lowest response rates included those related to patents associated with the firm (versus the respondent themselves), which are not used in this paper’s analyses.

CIC office space covers over 400,000 square feet across the locations. Average client tenure of current residents at CIC is 2.75 y, with the highest average in Cambridge. The largest space is One Broadway in Cambridge. The typical CIC firm is relatively small. St. Louis houses larger firms, on average, and the numbers for Cambridge and Boston reflect more use of coworking spaces, which tend to house the smallest companies and single-person firms.

While CIC does not systematically collect demographic information on their clients, we can confirm that our sample aligns well on gender. An internal CIC study conducted in 2015 found 27.6% of leaders were women, which is very close to the 24.2% in our respondent sample. Also, a random sample by CIC of 5% of clients in 2017 yielded 35% women, compared to 40.2% in our sample.

The survey first required respondents to categorize themselves as an employee, founder and/or CEO, owner, or other (e.g., board member, advisor). Our survey included extensive questions about the background of individuals, the traits of their firms, their networking behavior, their expectations for their company’s future, and their personalities. The survey instrument is included in *SI Appendix*, and we do not include in this analysis those in the “other” category, due to their different types of interaction with the CIC firms.

We define 4 mutually exclusive and collectively exhaustive groups for our core analysis: 1) entrepreneurs, those with position as founder, CEO, or owner who self-identify in a subsequent question to be a founder; 2) nonfounder CEO/leader, those who report being a founder, CEO, or owner but do not report having been a founder; 3) inventor employees, those with positions as employees who report having personally filed for a patent; and 4) noninventor employees, those with position as employees who do not report having personally filed for a patent.

The ability to isolate nonfounder CEOs/leaders is an important contribution of our sample. The development and scaling processes of ventures rely on hiring seasoned business leaders who can build the repeatable processes and operational backbone to refine emerging ideas and product prototypes and ultimately produce at scale. Often, venture funding is raised under the condition that the venture bring in these types of talents, and the funding (as well as location at CIC) can help facilitate this growth of a professional team.

The inventor group is also very important to study in the CIC context, especially given its outgrowth and proximity to MIT. The high-growth ventures housed in CIC often combine technology advances due to invention with the commercial insights that require entrepreneurial and business skill. Many CIC firms target patented inventions as a core development milestone, and inventors are held in high regard. This culture pulls from the original MIT-based ethos and continued spillovers from proximity to leading universities (an important placement criterion for all CIC facilities), the role of patents in venture fund raising, and similar venture objectives.

Many large companies also focus their CIC-based facilities on technology-intensive applications that often yield recognized inventions. Relative to the typical coworking space like WeWork, CIC-based operations tend to perform software coding as a stepping stone to products and applications that are frequently patented. Moreover, the most innovative software developments will be patented in their own right.

For a sample of venture heads, CIC facilitated linking individuals to external inventor data. For these matched individuals, 81% of inventors had filed patents with their current CIC venture; 19% had filed all of their patents prior to the founding of their current firm (although it could still be the case that those patents contributed to the founding of the CIC-based firm). In 38% of cases, the inventor had patents both before and then with their current CIC firm. In 43% of cases, the inventor’s only patents were after the founding of their current CIC venture.

*SI Appendix*, Tables S2 and S3a−S3d describe our core analytical sample. This sample includes 999 respondents who fell into one of the above 4 roles and completed at least the first risk tolerance question that commenced the personality section of the survey. Answering this question was a strong predictor for finishing the survey.

The sample composition is 18.5% entrepreneur, 13.5% nonfounder CEO/leader, 11.8% inventor employee, and 56.2% noninventor employees. While some entrepreneurs and nonfounder CEOs/leaders have filed patents (33% and 24% report doing so, respectively), our data and analyses strongly indicate that the entrepreneurial role is the stronger and more distinctive feature for the personality dimensions considered below. Thus, our core analysis commences with the inventor trait being only distinguished among employees, and we later discuss alternative designs with respect to the entrepreneur and nonfounder CEO/leader roles.

The core sample is about 60% male, 62% aged 25 to 44 y, and 19% doctorate holders. Entrepreneurs and nonfounder CEOs/leaders are typically over 35 y old, male, white, highly educated, and with degrees in business and economics. Employees tend to be younger, less educated, more in science, technology, engineering, and mathematics (STEM) fields, and more likely to be women. Inventor employees are predominantly men, immigrants, Asians, advanced degree holders, and STEM majors.

The survey measured risk attitudes in 3 ways. Respondents ranked themselves for “How much do you typically enjoy taking risks?” A follow-on asked specifically about taking financial risks. Ten-point scales ranged from “not at all happy to take risks” to “very happy to take risks.”

Participants were also incentivized to complete the survey with a reward that captured risk attitudes: They chose between receiving a guaranteed $5 Amazon gift card and entering a lottery drawing for a $2,000 gift card of their choice. The participants were presented with the estimated number of lottery entrants, and the lottery’s expected value was $2. Self-reported risk tolerance predicts choosing the lottery; 71% of those rating 8 or higher for general risk tolerance opted into the lottery, compared to 59% of those rating 7 or lower. These shares are similar for financial risk, and all differences are statistically significant.

The survey measured Big-5 personality traits: 1) “Openness to experience” describes the breadth, depth, originality, and complexity of an individual’s mental and experimental life. 2) “Conscientiousness” describes socially prescribed impulse control that facilitates task- and goal-oriented behavior. 3) “Extraversion” implies an energetic approach toward the social and material world and includes traits such as sociability, activity, assertiveness, and positive emotionality. 4) “Agreeableness” contrasts a prosocial and communal orientation toward others with antagonism and includes traits such as altruism, tender-mindedness, trust, and modesty. 5) “Neuroticism*”* contrasts emotional stability and even-temperedness with negative emotionality, such as feeling anxious, nervous, sad, and tense.

The survey also measured 4 traits that the literature has found salient for entrepreneurs (3): 1) self-efficacy, a belief in one’s abilities to complete tasks and fill roles; 2) internal locus of control, which contrasts a belief that one’s own decisions control one’s life with a belief that one’s life is controlled by factors beyond one’s control; 3) need for achievement, an individual’s desire for significant accomplishment, mastering of skills, and attaining challenging goals; and 4) innovativeness, how individuals respond to new opportunities and experiences.

To quantify these traits, respondents ranked themselves from 1 (strongly disagree) to 5 (strongly agree) on statements such as “I am talkative” and “I have a forgiving nature.” These questions and their aggregation follow prior literature (3). *SI Appendix*, Table S4 documents specific questions. We then calculate traits through unweighted averages of responses to statements connected to each trait. Some questions were “reversed” (e.g., “I am not a very creative person” is negatively associated with innovativeness), and we reversed the responses prior to averaging. *SI Appendix*, Tables S5a and S5b summarize personality traits by position and among entrepreneurs by gender, serial entrepreneurship, and having filed a patent.

## Results

**Fig. 1.**
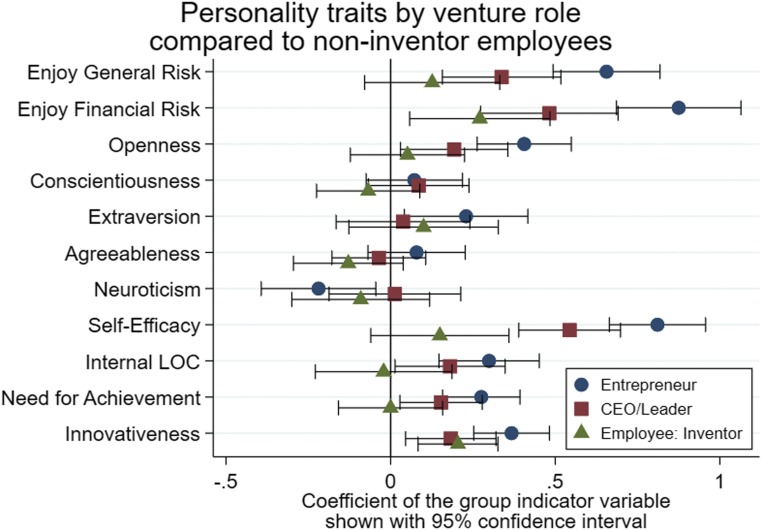
Regression results for personality traits of entrepreneurs, nonfounder CEOs/leaders, and inventor employees working at a CIC facility compared to noninventor employees. These categories are mutually exclusive and collectively exhaustive. Personality traits are measured on a 5-point scale, and the analysis reports coefficients and 95% confidence intervals for indicator variables indicating the respondent’s role. Regressions control for age, gender, ethnicity, immigration status, education level, education field, full-time status, and prior experience in the industry of individuals; *n* = 874 to 948. *SI Appendix*, Tables S6a–S6c report underlying regressions.

*SI Appendix*, Tables S6a–S6c report multivariate regressions of personality differences across roles that we summarize in Fig. 1. Regressions include indicator variables for the 3 reported roles of entrepreneurs, nonfounder CEOs/leaders, and inventor employees and compare the groups to the excluded category of noninventor employees. Estimations have 874 to 948 observations, with minor differences due to questions skipped. We control for an individual’s age, gender, ethnicity, immigration status, education level, education field, full-time status, and prior industry experience, using indicator variables. We transform the risk outcomes to a 5-point scale like other personality traits to allow easier comparability. We cluster SEs by firm.

Entrepreneurs display the highest self-reported risk tolerance, clearly distinguishable from inventor and noninventor employees. Entrepreneurs are also more tolerant of risk, with borderline statistical difference, from nonfounder CEOs/leaders. Nonfounder CEOs/leaders, in turn, report significant differences from noninventor employees but are less distinguishable from inventor employees.

Entrepreneurs are similar to noninventor employees for Big-5 personality traits, except for a tendency toward more openness and greater neuroticism. Nonfounder CEOs/leaders only show differences for openness from noninventor employees, and inventor employees show no meaningful differences from other employees. These limited differences align with earlier literature that has failed to consistently identify a strong entrepreneurial personality frame among the Big-5 traits.

Entrepreneurs do, however, stand out from noninventor employees for self-efficacy, internal locus of control, need for achievement, and innovativeness. Nonfounder CEOs/leaders are also statistically different from noninventor employees on all 4 dimensions, while less extreme than entrepreneurs. Interestingly, self-efficacy (the belief in one’s ability to complete tasks and roles) is the one dimension among these 4 personality traits on which some differences between entrepreneurs and nonfounder CEOs/leaders emerge. Inventor employees are only different from noninventor employees on the innovativeness metric.

**Fig. 2.**
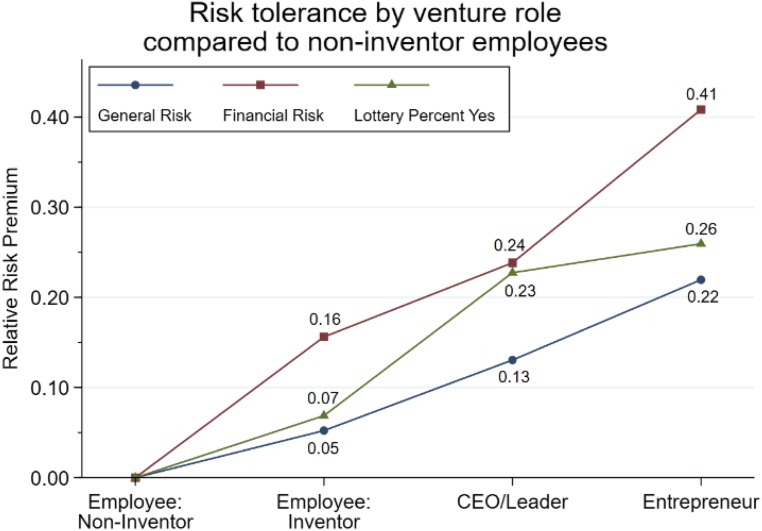
Percentage difference in risk tolerance of entrepreneurs, nonfounder CEOs/leaders, and inventor employees working at a CIC facility compared to noninventor employees. General Risk and Financial Risk metrics are built through individual’s responses to survey questions. The Lottery metric captures the propensity of individuals to select entering a lottery rather than accepting a fixed gift card as a reward for completing the survey; *n* = 874 to 948.

Fig. 2 continues the dimension of risk tolerance, showing variation from noninventor employees to entrepreneurs on the 3 risk dimensions. Entrepreneurs display a 22 to 41% premium over noninventor employees, compared to 13 to 24% for nonfounder CEOs/leaders. The premium for inventor employees is 5 to 16%. The consistent rank ordering and comparable magnitudes are remarkable given the quite different questions and the observational assessment via the lottery. Tabulations in *SI Appendix*, Table S5b also show that separate serial entrepreneurs with 2 or more ventures report greater risk tolerance than first-time entrepreneurs.

These documentations of variations in risk tolerance among respondents working in the same innovation center and local environment contribute to the literature. Cross-sectional data, however, cannot parse higher risk-taking due to past start-up efforts or business success from a stable long-term personality trait independent of career outcomes. For example, taking on the risks of founding and building an early-stage venture could increase entrepreneur’s risk tolerance relative to that of their employees.

Similarly, as 2 of the 3 risk measures are self-reported, there is scope for inflated perceptions by entrepreneurs of their risk-taking, perhaps due to the stereotypical ideal of entrepreneurs as risk-takers (akin to a social desirability response bias). Some evidence for this exists in that the premium for entrepreneurs over nonfounder CEOs/leaders is weakest with respect to taking the lottery, a behavioral measure of risk preference, compared to the self-reported measures. That both groups are consistently different from noninventor employees on both types of assessments provides assurances that inflated perceptions are not the sole driver of these outcomes, but interpretation should be cautious with respect to different career stages of respondents.

## Extensions

*SI Appendix*, Tables S7a–S7c show 2 important robustness checks on sample design. First, our sample includes some teams working with larger companies, and much of the entrepreneurial narrative would appear more suited for start-ups. This distinction can be hard to draw in an environment like CIC. For example, some start-ups at CIC get acquired by a large company but then continue to operate independently there. In other cases, a large company launches an independent venture at CIC to pursue an idea while operating like a start-up. As an analysis on this dimension, we show similar results when excluding respondents working at organizations with more than 25 employees; we also find similar robustness when just excluding the smaller number of CIC respondents who are working at public companies.

A second robustness considers variations on the nonfounder CEOs/leaders dimension. We can mostly isolate the test to nonfounder CEOs (versus other leaders) by excluding those who declared themselves “Owner” at the first question, thereby only focusing on those declaring “founder or CEO.” These results look, overall, quite similar, with the most noticeable exception being that the nonfounder CEO group is no longer statistically different from the noninventor employee group in terms of entering the lottery.

In *SI Appendix*, Tables S8a–S8c, we separate, among entrepreneurs and nonfounder CEOs/leaders, those who have past inventions versus those who do not. Among inventors in our sample, 28% are entrepreneurs, 16% are nonfounder CEOs/leaders, and 56% are employees. For measures of risk tolerance, incorporating this extra level of detail does not yield strong additional insights. Among the other traits, there is some evidence that past invention is correlated with greater openness and innovativeness among entrepreneurs and nonfounder CEOs/leaders. Unreported analyses also suggest greater risk tolerance among entrepreneurs and nonfounder CEOs/leaders leading companies that raised more than $250,000 in venture financing, but, again, caution is warranted in interpreting these differences, due to survivorship biases and similar issues.

Our core results are also quite similar when controlling for past start-up experience and/or the specific CIC buildings and floors of respondents. We further find similar results when isolating within-firm variation. For this purpose, we restrict the sample to respondents who belong to firms that have both entrepreneurs and employees surveyed. Regressions that model the traits of a firm’s entrepreneur (or the average of them when more than 2 are present) do not have strong explanatory power for the traits of employees. Personality is more connected to occupation/role than to company. Indeed, we find little correlation between the traits of venture leaders and those of their employees on the personality dimensions we study.

Finally, we have considered the potential overlap between inventor status and having a STEM education (i.e., both being proxies for technical work). Seventy percent of inventor employees report having their highest degree in a STEM field. We have confirmed that the results we jointly estimate, using STEM degree as a control variable, are quite stable if focusing on just one trait or the other. We also do not find significant differences across inventor types when estimating separate inventor variables for those with and without STEM degrees. STEM degrees are a frequent input to becoming an inventor, but our analyses indicate we are best served focusing on the inventor activity directly in order to capture the inputs into the successful CIC venture.

## Discussion

Research has struggled to agree on an “entrepreneurial personality,” given the vast heterogeneity among entrepreneurs (3, 4). We confirm that Big-5 traits are mostly similar across key roles in high-growth firms, with the biggest differences being toward greater openness among entrepreneurs and nonfounder CEOs/leaders. The more notable differences exist for self-efficacy, internal locus of control, need for achievement, and innovativeness. Self-reported tolerances for risk are also consistently greater for entrepreneurs than others, and they are mostly backed up in our behavioral assessment (5–7). This has implications for sorting by individuals into entrepreneurship (8–13) and the allocation of talent in society (14–16).

An important next step is to analyze whether these personality traits of entrepreneurs, leaders, and their teams can predict venture success. The CIC setting has potential to do this through follow-on surveys and/or the measurement of venture survival and employment growth as time accumulates. It will also be important to consider how personality traits shape the degree to which entrepreneurs and leaders utilize the resources available at CIC and/or receive spillover benefits from peers. Finally, as CIC expands to other countries, cross-country comparisons of these traits will be informative for understanding how the personality of entrepreneurs and their team members differ internationally.

### Data Availability.

An anonymized dataset and program that produces this paper’s results are available at https://doi.org/10.7910/DVN/XOR7VV.

## Supplementary Material

Supplementary File
