# Supplementary material for: Risk attitudes and personality traits of entrepreneurs and venture team members
Source: Proc Natl Acad Sci U S A. 2019 Aug 19;116(36):17712–6. doi: 10.1073/pnas.1908375116 (PMC6731656; doi:10.1073/pnas.1908375116)
Supplement: Supplementary File [file pnas.1908375116.sapp.pdf]

PNAS
www.pnas.org

Supplementary Information for

Risk Attitudes and Personality Traits of Entrepreneurs and Venture Team Members

Sari Pekkala Kerr, William R. Kerr, Margaret Dalton

William Kerr
Email: wkerr@hbs.edu

This PDF file includes:

Tables S1 to S8c
Survey Instrument

www.pnas.org/cgi/doi/10.1073/pnas.1908375116

Table S1. Descriptive statistics for CIC locations

	All	50 Milk	One Broadway	101 Main	St. Louis
**Year opened**		2014	2001	2012	2014
**Individuals**	5,645	1,236	2,467	464	1,478
Heads	1,168	346	577	59	186
non-Heads	4,477	890	1,890	405	1,292
**Total survey respondents**	1,222	199	493	86	348
Response rate	21.6	16.1	20.0	18.5	23.5
**Footprint (sq. ft.)**	422,177	93,410	155,147	52,465	121,155
**Average firm tenure at CIC in years**	2.8	2.3	4.4	4.4	1.6
**Average firm size at CIC in employees**	4.8	3.6	4.6	4.6	7.9
**Percent of firms that are nonprofits**	10.5	19.1	7.1	7.1	10.0

Note: One Broadway is the original CIC building at the edge of MIT. Boston-area expansions are 101 Main (one block away from One Broadway) and 50 Milk Street (Boston financial district).

Table S2. Descriptive statistics on core analysis sample by facility

	All	50 Milk	One Broadway	101 Main	St. Louis
**Core analysis sample**	999	182	443	78	293
Percent of sample		18.2	44.3	7.8	29.3
**Core analysis sample**					
Percent entrepreneurs	18.5	12.1	21.9	10.3	19.8
Percent non-founder CEOs/leaders	13.5	14.3	13.3	2.6	16.0
Percent inventor employees	11.8	7.7	15.8	9.0	9.2
Percent non-inventor employees	56.2	65.9	49.0	78.2	54.9
Percent aged < 25	8.9	11.4	8.8	10.3	7.2
Percent aged 25-34	37.3	37.5	35.8	47.4	36.4
Percent aged 35-44	24.3	29.0	21.0	26.9	25.8
Percent aged 45-54	17.1	13.6	21.9	6.4	14.8
Percent aged > 54	12.4	8.5	12.5	9.0	15.8
Percent women	40.1	44.3	38.4	42.9	39.0
Percent doctorate holders	18.9	8.2	25.3	11.5	17.8

Note: See Table S1. Core analysis sample restricted to those respondents classified into one of the four roles and who completed at least the first risk tolerance question that commenced the personality section of the survey.

Table S3a. Descriptive statistics on survey responses by individual's role

	All responses	Core sample	Entrepreneur	Non-founder CEO/leader	Inventor employee	Non-inventor employee
**Respondents**	1,222	999	185	135	118	561
**Percent of core sample**			18.5	13.5	11.8	56.2
**Female**	40.2	40.1	20.3	28.2	23.9	52.9
**Immigrant**	26.0	25.6	25.7	21.1	44.4	22.7
**Age**						
Under 25	8.7	8.9	2.8	3.0	1.7	13.8
25-34	37.2	37.3	17.3	24.8	30.8	48.2
35-44	24.5	24.3	24.0	30.8	31.6	21.2
45-54	17.3	17.1	28.5	23.3	23.1	10.7
Over 55	12.3	12.4	27.4	18.0	12.8	6.2
**Race and ethnicity**						
Asian	12.8	12.9	8.7	9.6	23.9	12.7
African American	3.5	3.6	5.4	5.2	0.0	3.4
Hispanic/Latino	5.5	5.5	5.4	4.4	4.3	6.1
White	73.5	73.8	74.5	75.6	67.5	74.6
Other responses	3.8	3.9	6.0	4.4	3.4	3.2
**Education**						
BA/MA	75.3	75.3	64.7	61.5	61.0	85.2
PhD	19.0	18.9	29.9	34.8	37.3	7.5
Other	5.7	5.8	5.4	3.7	1.7	7.3
**Field of Education**						
STEM	36.0	36.1	35.1	27.1	70.1	31.4
Business or economics	29.5	29.3	42.7	39.1	11.1	26.4
Other	34.6	34.6	22.2	33.8	18.8	42.2
**Experience**						
Prior work in industry	62.6	62.1	82.1	69.6	65.3	53.0
Prior work in a startup	47.8	47.4	89.7	23.0	61.0	36.4

Note: We segment the sample into entrepreneurs, non-founder CEOs/leaders, inventor employees, and non-inventor employees as described in the text. Race and ethnicity shares can add up to slightly more or less than 100% due to multiple boxes checked or none reported.

Table S3b. Prevalence of invention and STEM among Entrepreneurs

	Non-Inventor	Inventor	Non-STEM	STEM
**Respondents**	124	61	120	65
**Percent of sample**	67.0	33.0	64.9	35.1
**Female**	23.8	13.3	23.7	14.1
**Immigrant**	22.8	31.7	24.4	28.1
**Age**				
Under 25	3.4	1.7	1.7	4.8
25-34	20.2	11.7	15.5	20.6
35-44	24.4	23.3	22.4	27.0
45-54	26.9	31.7	33.6	19.0
Over 55	25.2	31.7	26.7	28.6
**Race and ethnicity**				
Asian	8.1	10.0	7.6	10.8
African American	8.1	0.0	8.4	0.0
Hispanic/Latino	5.6	5.0	6.7	3.1
White	72.6	78.3	73.1	76.9
Other responses	5.6	6.7	5.9	6.2
**Education**				
BA/MA	72.6	48.3	73.1	49.2
PhD	20.2	50.0	21.8	44.6
Other	7.3	1.7	5.0	6.2
**Field of Education**				
STEM	21.8	62.3	0.0	100.0
Business or economics	50.8	26.2	65.8	0.0
Other	27.4	11.5	34.2	0.0
**Experience**				
Prior work in industry	82.9	80.3	83.2	80.0
Prior work in a startup	87.1	95.1	88.3	92.3
Founded 1-2 firms	57.3	41.0	61.7	52.3
Founded 3+ firms	42.7	59.0	48.3	47.7

Note: See Table S3a.

Table S3c. Prevalence of invention and STEM among Non-founder CEOs/leaders

	Non-Inventor	Inventor	Non-STEM	STEM
**Respondents**	102	33	97	36
**Percent of sample**	75.6	24.4	71.9	26.7
**Female**	34.3	9.4	30.9	22.9
**Immigrant**	17.8	31.3	12.6	41.7
**Age**				
Under 25	3.0	3.1	1.1	8.3
25-34	23.8	28.1	24.2	25.0
35-44	33.7	21.9	34.7	22.2
45-54	23.8	21.9	21.1	27.8
Over 55	15.8	25.0	18.9	16.7
**Race and ethnicity**				
Asian	6.9	18.2	7.2	13.9
African American	6.9	0.0	6.2	2.8
Hispanic/Latino	3.9	6.1	2.1	11.1
White	76.5	72.7	77.3	72.2
Other responses	3.9	6.1	6.2	0.0
**Education**				
BA/MA	68.6	39.4	68.0	44.4
PhD	27.5	57.6	29.9	50.0
Other	3.9	3.0	2.1	5.6
**Field of Education**				
STEM	18.8	53.1	0.0	100.0
Business or economics	42.6	28.1	53.6	0.0
Other	38.6	18.8	46.4	0.0
**Experience**				
Prior work in industry	71.6	63.6	72.2	63.9
Prior work in a startup	17.6	39.4	22.7	22.2

Note: See Table S3a.

Table S3d. Prevalence of invention and STEM among Employees

	Non-Inventor	Inventor	Non-STEM	STEM
**Respondents**	561	118	414	256
**Percent of sample**	82.6	17.4	61.0	37.7
**Female**	52.9	23.9	55.9	34.4
**Immigrant**	22.7	44.4	20.5	37.0
**Age**				
Under 25	13.8	1.7	11.6	11.8
25-34	48.2	30.8	46.6	42.0
35-44	21.2	31.6	21.4	25.9
45-54	10.7	23.1	11.8	14.9
Over 55	6.2	12.8	8.6	5.5
**Race and ethnicity**				
Asian	12.7	23.9	10.0	22.7
African American	3.4	0.0	3.4	1.6
Hispanic/Latino	6.1	4.3	7.0	3.9
White	74.6	67.5	77.2	67.1
Other responses	3.2	3.4	2.7	3.9
**Education**				
BA/MA	85.2	61.0	89.4	68.4
PhD	7.5	37.3	5.1	25.4
Other	7.3	1.7	5.6	6.3
**Field of Education**				
STEM	31.4	70.1	0.0	100.0
Business or economics	26.4	11.1	38.3	0.0
Other	42.2	18.8	61.7	0.0
**Experience**				
Prior work in industry	53.0	65.3	51.2	60.5
Prior work in a startup	36.4	61.0	37.5	46.7

Note: See Table S3a.

Table S4. Survey questions used to calculate personality traits

Trait	Survey Questions	Reverse	Scale
	A. Risk		
Enjoy Risk	How much do you typically enjoy taking risks?		[1,10]
Financial Risk	Some activities involve a "financial" risk, such as starting a business, investing, or gambling and betting — that is, there is a risk of losing money or other assets. In general, what is your propensity for accepting financial risks?		[1,10]
Lottery	All respondents can either choose to receive a $5 Amazon gift card, or to participate in a drawing for a $2,000 gift card of choice. We expect to receive around 1000 participants in the drawing. Please make your choice: 1) Please send me a $5 Amazon gift card. 2) Please enter me in a drawing for the $2,000 gift card.		[1,2]
	B. Big 5		
Extraversion	I am talkative		[1,5]
	I am reserved	Yes	[1,5]
	I am social and outgoing		[1,5]
Conscientiousness	I am very thorough in my actions		[1,5]
	I am often lazy	Yes	[1,5]
	I do things efficiently		[1,5]
Openness	I am original, come up with new ideas		[1,5]
	I have an active imagination		[1,5]
	I value artistic, aesthetic experiences		[1,5]
Neuroticism	I am relaxed, handle stress well	Yes	[1,5]
	I get nervous easily and worry		[1,5]
Agreeableness	I have a forgiving nature		[1,5]
	I am kind and considerate to others		[1,5]
	C. Other Traits		
Self-Efficacy	If I work hard, I can successfully start a business		[1,5]
	Overall, my skills and abilities will help me start a business		[1,5]
	My past experience will be very valuable in starting a business		[1,5]
	I am confident I can put in the effort needed to start a business		[1,5]
Internal LOC	I believe that I am primarily responsible for my own successes and failures		[1,5]
Need for Achievement	I feel a great deal of pride when I complete a project successfully		[1,5]
	I have a desire to achieve positive results even if it requires a lot of additional effort		[1,5]
Innovativeness	I surprise people with my novel ideas		[1,5]
	People ask me for help in creative activities		[1,5]
	I get more satisfaction from mastering a skill than developing a new idea.		[1,5]
	I prefer work that requires original thinking		[1,5]
	I like a job which demands skill and practice rather than inventiveness	Yes	[1,5]
	I am not a very creative person	Yes	[1,5]

Table S5a: Personality traits by position

	Core sample	Entrepreneur	Non-founder CEO/leader	Inventor employee	Non-inventor employee
			A. Risk Tolerance		
Enjoy Risk	6.92	8.02	7.41	6.86	6.44
	(1.95)	(1.55)	(1.64)	(1.83)	(1.99)
Financial Risk	5.75	7.39	6.37	5.88	5.03
	(2.28)	(1.96)	(2.13)	(2.01)	(2.14)
Opt into Lottery (% yes)	63.43	73.65	71.77	62.50	58.48
	(48.19)	(44.14)	(45.19)	(48.62)	(49.32)
			B. Big 5 Traits		
Openness	3.88	4.15	3.96	3.86	3.79
	(0.73)	(0.72)	(0.75)	(0.72)	(0.71)
Conscientiousness	3.90	3.92	3.93	3.79	3.91
	(0.68)	(0.75)	(0.72)	(0.66)	(0.65)
Extraversion	3.37	3.51	3.36	3.25	3.35
	(0.97)	(0.93)	(1.00)	(0.92)	(0.98)
Agreeableness	4.02	4.05	3.98	3.88	4.06
	(0.75)	(0.76)	(0.71)	(0.82)	(0.72)
Neuroticism	2.54	2.26	2.50	2.44	2.66
	(0.91)	(0.86)	(0.91)	(0.85)	(0.90)
			C. Other Traits		
Self-efficacy	3.96	4.59	4.32	3.90	3.67
	(0.90)	(0.57)	(0.65)	(0.85)	(0.92)
Internal Locus of Control	4.30	4.56	4.40	4.16	4.22
	(0.80)	(0.70)	(0.76)	(0.90)	(0.79)
Need for Achievement	4.47	4.65	4.52	4.37	4.42
	(0.61)	(0.57)	(0.60)	(0.74)	(0.58)
Innovativeness	3.52	3.79	3.63	3.67	3.37
	(0.63)	(0.57)	(0.63)	(0.57)	(0.62)

Sample: See Table S3.

Table S5b: Personality traits by position

	Male entrepreneurs	Female entrepreneurs	Entrepreneur with 1-2 firms	Entrepreneurs with 3+ firms	Inventor entrepreneurs	Non-inventor entrepreneurs
			A. Risk Tolerance			
Enjoy Risk	8.14	7.57	7.73	8.34	8.13	7.97
	(1.46)	(1.85)	(1.57)	(1.48)	(1.54)	(1.56)
Financial Risk	7.57	6.67	6.96	7.85	7.36	7.40
	(1.85)	(2.26)	(2.06)	(1.75)	(1.91)	(2.00)
Opt into Lottery (% yes)	74.81	66.67	72.41	75.00	70.91	75.00
	(43.58)	(47.87)	(44.95)	(43.57)	(45.84)	(43.50)
			B. Big 5 Traits			
Openness	4.12	4.26	4.11	4.20	4.21	4.12
	(0.71)	(0.77)	(0.77)	(0.65)	(0.73)	(0.71)
Conscientiousness	3.90	4.06	3.85	4.00	3.91	3.93
	(0.75)	(0.73)	(0.74)	(0.75)	(0.85)	(0.70)
Extraversion	3.49	3.52	3.47	3.55	3.39	3.56
	(0.93)	(0.94)	(0.93)	(0.93)	(1.01)	(0.89)
Agreeableness	4.03	4.17	4.11	3.98	4.01	4.07
	(0.74)	(0.80)	(0.73)	(0.79)	(0.72)	(0.78)
Neuroticism	2.16	2.61	2.40	2.10	2.16	2.31
	(0.80)	(0.98)	(0.88)	(0.83)	(0.81)	(0.89)
			C. Other Traits			
Self-efficacy	4.61	4.53	4.50	4.70	4.56	4.61
	(0.56)	(0.57)	(0.66)	(0.43)	(0.68)	(0.51)
Internal Locus of Control	4.54	4.61	4.51	4.62	4.50	4.59
	(0.73)	(0.60)	(0.78)	(0.60)	(0.81)	(0.64)
Need for Achievement	4.63	4.71	4.62	4.68	4.65	4.65
	(0.59)	(0.48)	(0.61)	(0.52)	(0.66)	(0.52)
Innovativeness	3.80	3.74	3.70	3.90	3.99	3.69
	(0.56)	(0.63)	(0.61)	(0.50)	(0.53)	(0.57)

Sample: See Table S5a.

Table S6a. Multivariate regressions for risk tolerance

	Enjoy general risk, measured on 5-point scale	Enjoy financial risk, measured on 5-point scale	Entered the lottery rather than taking gift card
Entrepreneur	0.648***	0.863***	0.115**
	(0.0822)	(0.0961)	(0.0489)
Non-founder CEO/leader	0.330***	0.471***	0.0927*
	(0.0913)	(0.106)	(0.0534)
Inventor employee	0.121	0.262**	0.0502
	(0.104)	(0.108)	(0.0519)
Female	-0.258***	-0.472***	-0.00482
	(0.0667)	(0.0736)	(0.0355)
Fulltime with company	-0.0181	-0.0167	-0.00255
	(0.0947)	(0.118)	(0.0530)
Young (Under 35 years old)	-0.0166	-0.0558	-0.0920**
	(0.0663)	(0.0803)	(0.0375)
Immigrant	0.0851	0.244***	0.0212
	(0.0701)	(0.0767)	(0.0405)
Doctorate degree	-0.0142	0.292*	0.0591
	(0.173)	(0.173)	(0.0796)
Bachelors or masters degree	-0.0423	0.198	0.0278
	(0.154)	(0.167)	(0.0752)
White	0.000372	0.128	0.0924**
	(0.0687)	(0.0830)	(0.0437)
Highest degree in STEM field	0.00670	0.0971	-0.0894**
	(0.0776)	(0.0757)	(0.0408)
Highest degree in business/econ	0.173**	0.371***	-0.0241
	(0.0779)	(0.0862)	(0.0396)
Prior industry experience	-0.0154	-0.0560	-0.0123
	(0.0683)	(0.0732)	(0.0349)
Constant	3.639***	2.640***	0.581***
	(0.203)	(0.203)	(0.0990)
Observations	948	945	874
R-squared	0.126	0.230	0.040

Note: All explanatory variables take the form of indicator variables. Indicators for entrepreneur, non-founder CEOs/leaders, and inventor employees are measured relative to non-inventor employees. Standard errors are clustered by firm. *** p<0.01, ** p<0.05, * p<0.1

Table S6b. Multivariate regressions for Big-5 traits

	Openness, measured on 5-point scale	Conscientiousness, measured on 5-point scale	Extraversion, measured on 5-point scale	Agreeableness, measured on 5-point scale	Neuroticism, measured on 5-point scale
Entrepreneur	0.412***	0.0696	0.220**	0.0871	-0.238***
	(0.0723)	(0.0740)	(0.0949)	(0.0758)	(0.0891)
Non-founder CEO/leader	0.199**	0.0831	0.0306	-0.0233	-0.00499
	(0.0828)	(0.0777)	(0.103)	(0.0730)	(0.102)
Inventor employee	0.0553	-0.0684	0.0957	-0.120	-0.105
	(0.0880)	(0.0797)	(0.115)	(0.0849)	(0.107)
Female	0.000124	0.227***	0.160**	0.120**	0.313***
	(0.0539)	(0.0485)	(0.0657)	(0.0531)	(0.0646)
Fulltime with company	0.134	0.0164	0.0163	0.0919	0.0610
	(0.0865)	(0.0720)	(0.0898)	(0.0860)	(0.0970)
Young (Under 35 years old)	-0.00718	0.0193	-0.0651	0.0483	0.171**
	(0.0543)	(0.0459)	(0.0691)	(0.0541)	(0.0688)
Immigrant	-0.0198	0.0435	0.0642	-0.0414	-0.0724
	(0.0560)	(0.0512)	(0.0771)	(0.0581)	(0.0726)
Doctorate degree	0.0134	0.0362	-0.182	0.140	0.0288
	(0.111)	(0.120)	(0.152)	(0.137)	(0.132)
Bachelors or masters degree	-0.0217	-0.0115	-0.266*	0.107	0.103
	(0.109)	(0.105)	(0.142)	(0.128)	(0.119)
White	0.0257	0.0571	-0.0186	-0.0250	0.00139
	(0.0544)	(0.0516)	(0.0741)	(0.0589)	(0.0697)
Highest degree in STEM field	-0.118**	-0.0121	-0.411***	-0.122*	-0.00472
	(0.0584)	(0.0590)	(0.0840)	(0.0642)	(0.0754)
Highest degree in business/econ	-0.273***	-0.0143	-0.0142	-0.124*	-0.0959
	(0.0670)	(0.0620)	(0.0839)	(0.0715)	(0.0832)
Prior industry experience	-0.0386	0.0858*	-0.153**	-0.0487	0.0171
	(0.0546)	(0.0466)	(0.0687)	(0.0515)	(0.0656)
Constant	3.817***	3.666***	3.724***	3.905***	2.302***
	(0.137)	(0.132)	(0.169)	(0.159)	(0.164)
Observations	911	914	913	915	916
R-squared	0.062	0.033	0.060	0.026	0.077

Note: See Table S6a.

Table S6c. Multivariate regressions for additional entrepreneurial traits

	Self efficacy, measured on 5-point scale	Internal LOC, measured on 5-point scale	Need for Achievement, on 5-point scale	Innovativeness, measured on 5-point scale
Entrepreneur	0.816***	0.310***	0.289***	0.374***
	(0.0737)	(0.0770)	(0.0599)	(0.0581)
Non-founder CEO/leader	0.548***	0.189**	0.164**	0.187***
	(0.0783)	(0.0849)	(0.0638)	(0.0697)
Inventor employee	0.153	-0.0138	0.00948	0.208***
	(0.106)	(0.106)	(0.0808)	(0.0616)
Female	-0.160**	0.0764	0.148***	-0.130***
	(0.0619)	(0.0578)	(0.0424)	(0.0463)
Fulltime with company	-0.108	0.0554	0.0857	0.0897
	(0.0828)	(0.0856)	(0.0667)	(0.0670)
Young (Under 35 years old)	0.0520	-0.112**	-0.0478	-0.0267
	(0.0690)	(0.0567)	(0.0450)	(0.0446)
Immigrant	0.0736	0.0256	-0.0221	-0.0256
	(0.0603)	(0.0645)	(0.0527)	(0.0483)
Doctorate degree	0.315*	-0.174	-0.110	0.258***
	(0.175)	(0.122)	(0.0969)	(0.0997)
Bachelors or masters degree	0.182	-0.114	-0.0280	0.141
	(0.174)	(0.105)	(0.0809)	(0.0959)
White	-0.0191	0.0663	0.0653	0.0885*
	(0.0638)	(0.0598)	(0.0540)	(0.0517)
Highest degree in STEM field	0.0454	0.0775	0.0317	-0.0418
	(0.0674)	(0.0689)	(0.0521)	(0.0488)
Highest degree in business/econ	0.197**	0.104	-0.0639	-0.127**
	(0.0804)	(0.0679)	(0.0555)	(0.0562)
Prior industry experience	0.0605	0.0225	-0.00569	-0.0360
	(0.0610)	(0.0620)	(0.0430)	(0.0438)
Constant	3.530***	4.183***	4.284***	3.255***
	(0.197)	(0.146)	(0.113)	(0.114)
Observations	911	916	915	905
R-squared	0.196	0.043	0.046	0.107

Note: See Table S6a.

Table S7a. Extensions on Table S6a

	Enjoy general risk, measured on 5-point scale	Enjoy financial risk, measured on 5-point scale	Entered the lottery rather than taking gift card
	A. Baseline outcomes		
Entrepreneur	0.648***	0.863***	0.115**
	(0.0822)	(0.0961)	(0.0489)
Non-founder CEO/leader	0.330***	0.471***	0.0927*
	(0.0913)	(0.106)	(0.0534)
Inventor employee	0.121	0.262**	0.0502
	(0.104)	(0.108)	(0.0519)
Observations	948	945	874
R-squared	0.126	0.230	0.040
	B. Sample excluding firms with 25+ employees		
Entrepreneur	0.653***	0.882***	0.139**
	(0.0985)	(0.116)	(0.0566)
Non-founder CEO/leader	0.401***	0.486***	0.103*
	(0.107)	(0.125)	(0.0604)
Inventor employee	0.0961	0.240*	0.0826
	(0.117)	(0.131)	(0.0673)
Observations	657	656	602
R-squared	0.158	0.241	0.030
	C. Sample focusing on non-founder CEOs		
Entrepreneur	0.640***	0.907***	0.117**
	(0.0874)	(0.100)	(0.0516)
Non-founder CEO	0.376***	0.513***	0.0843
	(0.0964)	(0.117)	(0.0577)
Inventor employee	0.117	0.270**	0.0481
	(0.105)	(0.109)	(0.0520)
Observations	894	891	822
R-squared	0.121	0.235	0.042

Note: See Table S6a. Estimations include unreported explanatory variables.

Table S7b. Extensions on Table S6b

	Openness, measured on 5-point scale	Conscientiousness, measured on 5-point scale	Extraversion, measured on 5-point scale	Agreeableness, measured on 5-point scale	Neuroticism, measured on 5-point scale
	A. Baseline outcomes				
Entrepreneur	0.412***	0.0696	0.220**	0.0871	-0.238***
	(0.0723)	(0.0740)	(0.0949)	(0.0758)	(0.0891)
Non-founder CEO/leader	0.199**	0.0831	0.0306	-0.0233	-0.00499
	(0.0828)	(0.0777)	(0.103)	(0.0730)	(0.102)
Inventor employee	0.0553	-0.0684	0.0957	-0.120	-0.105
	(0.0880)	(0.0797)	(0.115)	(0.0849)	(0.107)
Observations	911	914	913	915	916
R-squared	0.062	0.033	0.060	0.026	0.077
	B. Sample excluding firms with 25+ employees				
Entrepreneur	0.501***	0.0850	0.287**	0.0846	-0.214**
	(0.0812)	(0.0824)	(0.114)	(0.0902)	(0.108)
Non-founder CEO/leader	0.279***	0.0985	0.00766	0.0150	-0.0159
	(0.0895)	(0.0862)	(0.120)	(0.0842)	(0.114)
Inventor employee	0.119	0.0128	0.0966	-0.0267	-0.176
	(0.106)	(0.0892)	(0.140)	(0.113)	(0.134)
Observations	630	632	631	633	634
R-squared	0.088	0.039	0.085	0.025	0.079
	C. Sample focusing on non-founder CEOs				
Entrepreneur	0.387***	0.0698	0.192*	0.0679	-0.265***
	(0.0795)	(0.0767)	(0.101)	(0.0801)	(0.0943)
Non-founder CEO	0.253***	0.106	0.0942	-0.0124	0.0156
	(0.0830)	(0.0834)	(0.112)	(0.0782)	(0.111)
Inventor employee	0.0544	-0.0638	0.0801	-0.128	-0.0942
	(0.0886)	(0.0797)	(0.118)	(0.0855)	(0.107)
Observations	856	859	858	860	862
R-squared	0.059	0.032	0.056	0.025	0.086

Note: See Table S6a. Estimations include unreported explanatory variables.

Table S7c. Extensions on Table S6c

	Self efficacy, measured on 5-point scale	Internal LOC, measured on 5-point scale	Need for Achievement, on 5-point scale	Innovativeness, measured on 5-point scale
	A. Baseline outcomes			
Entrepreneur	0.816***	0.310***	0.289***	0.374***
	(0.0737)	(0.0770)	(0.0599)	(0.0581)
Non-founder CEO/leader	0.548***	0.189**	0.164**	0.187***
	(0.0783)	(0.0849)	(0.0638)	(0.0697)
Inventor employee	0.153	-0.0138	0.00948	0.208***
	(0.106)	(0.106)	(0.0808)	(0.0616)
Observations	911	916	915	905
R-squared	0.196	0.043	0.046	0.107
	B. Sample excluding firms with 25+ employees			
Entrepreneur	0.818***	0.295***	0.339***	0.371***
	(0.0818)	(0.0922)	(0.0699)	(0.0677)
Non-founder CEO/leader	0.558***	0.232**	0.241***	0.213***
	(0.0857)	(0.0956)	(0.0715)	(0.0778)
Inventor employee	0.0890	-0.123	0.0639	0.217***
	(0.122)	(0.138)	(0.106)	(0.0759)
Observations	629	634	633	624
R-squared	0.204	0.048	0.056	0.131
	C. Sample focusing on non-founder CEOs			
Entrepreneur	0.794***	0.304***	0.263***	0.366***
	(0.0807)	(0.0819)	(0.0651)	(0.0610)
Non-founder CEO	0.547***	0.152*	0.148**	0.249***
	(0.0862)	(0.0914)	(0.0669)	(0.0766)
Inventor employee	0.142	-0.0320	-0.00481	0.204***
	(0.108)	(0.106)	(0.0807)	(0.0622)
Observations	856	861	860	851
R-squared	0.185	0.039	0.042	0.112

Note: See Table S6a. Estimations include unreported explanatory variables.

Table S8a. Table S6a with inventor breakouts for non-employees

	Enjoy general risk, measured on 5-point scale	Enjoy financial risk, measured on 5-point scale	Entered the lottery rather than taking gift card
Entrepreneur: Inventor	0.744***	0.846***	0.0923
	(0.114)	(0.138)	(0.0722)
Entrepreneur: Non-Inventor	0.609***	0.872***	0.125**
	(0.0898)	(0.109)	(0.0560)
Non-founder CEO/leader: Inventor	0.440***	0.536***	0.103
	(0.142)	(0.184)	(0.0909)
Non-founder CEO/leader: Non-Inventor	0.302***	0.452***	0.0892
	(0.102)	(0.118)	(0.0569)
Employee: Inventor	0.133	0.264**	0.0489
	(0.105)	(0.109)	(0.0520)
Female	-0.254***	-0.471***	-0.00483
	(0.0668)	(0.0737)	(0.0355)
Fulltime with company	-0.0134	-0.0175	-0.00387
	(0.0946)	(0.119)	(0.0534)
Young (Under 35 years old)	-0.0151	-0.0572	-0.0929**
	(0.0667)	(0.0805)	(0.0377)
Immigrant	0.0814	0.243***	0.0211
	(0.0702)	(0.0773)	(0.0406)
Doctorate degree	-0.0360	0.290*	0.0610
	(0.173)	(0.173)	(0.0805)
Bachelors or masters degree	-0.0498	0.198	0.0289
	(0.154)	(0.167)	(0.0754)
White	-0.00377	0.128	0.0929**
	(0.0689)	(0.0834)	(0.0438)
Highest degree in STEM field	-0.00703	0.0961	-0.0877**
	(0.0786)	(0.0760)	(0.0413)
Highest degree in business/econ	0.174**	0.370***	-0.0247
	(0.0778)	(0.0864)	(0.0397)
Prior industry experience	-0.0123	-0.0554	-0.0127
	(0.0683)	(0.0730)	(0.0351)
Constant	3.647***	2.641***	0.581***
	(0.203)	(0.203)	(0.0992)
Observations	948	945	874
R-squared	0.127	0.230	0.040

Note: See Table S6a. Indicators for roles are measured relative to non-inventor employees. Standard errors are clustered by firm. *** p<0.01, ** p<0.05, * p<0.1

Table S8b. Table S6b with inventor breakouts for non-employees

	Openness, measured on 5-point scale	Conscientiousness, measured on 5-point scale	Extraversion, measured on 5-point scale	Agreeableness, measured on 5-point scale	Neuroticism, measured on 5-point scale
Entrepreneur: Inventor	0.518***	0.0451	0.240	0.0303	-0.298**
	(0.118)	(0.131)	(0.163)	(0.117)	(0.127)
Entrepreneur: Non-Inventor	0.373***	0.0824	0.216**	0.111	-0.213**
	(0.0781)	(0.0812)	(0.0994)	(0.0856)	(0.101)
Non-founder CEO/leader: Inventor	0.576***	0.187	0.245	-0.0976	-0.0747
	(0.120)	(0.128)	(0.176)	(0.145)	(0.168)
Non-founder CEO/leader: Non-Inventor	0.0992	0.0543	-0.0269	-0.00488	0.0126
	(0.0911)	(0.0864)	(0.115)	(0.0787)	(0.110)
Employee: Inventor	0.0773	-0.0666	0.105	-0.127	-0.113
	(0.0888)	(0.0804)	(0.116)	(0.0864)	(0.107)
Female	0.0111	0.230***	0.166**	0.118**	0.310***
	(0.0541)	(0.0491)	(0.0659)	(0.0533)	(0.0650)
Fulltime with company	0.140	0.0154	0.0177	0.0890	0.0580
	(0.0866)	(0.0731)	(0.0890)	(0.0859)	(0.0971)
Young (Under 35 years old)	-0.00968	0.0171	-0.0677	0.0476	0.170**
	(0.0542)	(0.0459)	(0.0693)	(0.0539)	(0.0688)
Immigrant	-0.0288	0.0418	0.0598	-0.0392	-0.0701
	(0.0561)	(0.0517)	(0.0772)	(0.0589)	(0.0731)
Doctorate degree	-0.0281	0.0331	-0.199	0.153	0.0428
	(0.113)	(0.121)	(0.153)	(0.139)	(0.134)
Bachelors or masters degree	-0.0355	-0.0117	-0.271*	0.112	0.109
	(0.109)	(0.105)	(0.142)	(0.129)	(0.119)
White	0.0203	0.0575	-0.0202	-0.0226	0.00399
	(0.0547)	(0.0516)	(0.0749)	(0.0591)	(0.0702)
Highest degree in STEM field	-0.146**	-0.0144	-0.423***	-0.113*	0.00408
	(0.0610)	(0.0603)	(0.0854)	(0.0658)	(0.0754)
Highest degree in business/econ	-0.277***	-0.0164	-0.0170	-0.124*	-0.0961
	(0.0667)	(0.0618)	(0.0841)	(0.0715)	(0.0833)
Prior industry experience	-0.0312	0.0869*	-0.149**	-0.0508	0.0151
	(0.0542)	(0.0468)	(0.0686)	(0.0516)	(0.0656)
Constant	3.835***	3.669***	3.732***	3.900***	2.297***
	(0.138)	(0.132)	(0.169)	(0.160)	(0.164)
Observations	911	914	913	915	916
R-squared	0.073	0.034	0.061	0.027	0.077

Note: See Table S8a.

Table S8c. Table S6c with inventor breakouts for non-employees

	Self efficacy, measured on 5-point scale	Internal LOC, measured on 5-point scale	Need for Achievement, on 5-point scale	Innovativeness, measured on 5-point scale
Entrepreneur: Inventor	0.747***	0.280**	0.305***	0.586***
	(0.119)	(0.133)	(0.107)	(0.0876)
Entrepreneur: Non-Inventor	0.847***	0.325***	0.282***	0.288***
	(0.0772)	(0.0801)	(0.0631)	(0.0638)
Non-founder CEO/leader: Inventor	0.571***	0.247*	0.147	0.509***
	(0.133)	(0.135)	(0.120)	(0.125)
Non-founder CEO/leader: Non-Inventor	0.539***	0.171*	0.169**	0.109
	(0.0836)	(0.0940)	(0.0691)	(0.0737)
Employee: Inventor	0.149	-0.0140	0.0101	0.236***
	(0.108)	(0.107)	(0.0821)	(0.0620)
Female	-0.160**	0.0779	0.147***	-0.120**
	(0.0620)	(0.0579)	(0.0427)	(0.0466)
Fulltime with company	-0.112	0.0539	0.0864	0.102
	(0.0837)	(0.0859)	(0.0671)	(0.0664)
Young (Under 35 years old)	0.0496	-0.114**	-0.0471	-0.0241
	(0.0693)	(0.0569)	(0.0450)	(0.0441)
Immigrant	0.0742	0.0250	-0.0220	-0.0334
	(0.0606)	(0.0647)	(0.0532)	(0.0477)
Doctorate degree	0.324*	-0.173	-0.111	0.204**
	(0.177)	(0.126)	(0.100)	(0.101)
Bachelors or masters degree	0.186	-0.113	-0.0287	0.122
	(0.174)	(0.105)	(0.0813)	(0.0966)
White	-0.0168	0.0671	0.0649	0.0774
	(0.0640)	(0.0601)	(0.0542)	(0.0513)
Highest degree in STEM field	0.0513	0.0781	0.0309	-0.0746
	(0.0707)	(0.0715)	(0.0541)	(0.0499)
Highest degree in business/econ	0.195**	0.102	-0.0633	-0.126**
	(0.0802)	(0.0677)	(0.0553)	(0.0557)
Prior industry experience	0.0594	0.0228	-0.00565	-0.0273
	(0.0616)	(0.0622)	(0.0432)	(0.0434)
Constant	3.527***	4.183***	4.284***	3.274***
	(0.197)	(0.147)	(0.114)	(0.115)
Observations	911	916	915	905
R-squared	0.197	0.044	0.046	0.124

Note: See Table S8a.

Survey Instrument

Note: This has been edited to include only questions relevant to this analysis. It has been indicated below where questions have been removed. For a full version of the survey instrument, please see Kerr & Kerr (2018) or contact wkerr@hbs.edu.

1. Please characterize your position in the company you are most involved with at CIC.
This question requires an answer in order to start the survey
- o Employee (1)
- o Founder and/or CEO (2)
- o Owner (3)
- o Other (e.g. board member, advisor) (4)

2. Is this position full-time or part-time?
- ❍ Full-time (1)
- ❍ Part-time (2)

Experience with CIC
3. How long have you cumulatively been a client at CIC?
- ❍ < 6 months (1)
- ❍ 6-18 months (2)
- ❍ 18-36 months (3)
- ❍ 3-5 years (4)
- ❍ 5+ years (5)

4 How long do you plan to stay at CIC?
- ❍ < 6 months (1)
- ❍ 6-18 months (2)
- ❍ 18-36 months (3)
- ❍ 3-5 years (4)
- ❍ 5+ years (5)

<u>Display the following questions for clients in MA (Cambridge and Boston)</u>
5. Which building are you currently located in?
- ❍ 50 Milk Street (1)
- ❍ 1 Broadway (2)
- ❍ 101 Main Street (3)

Display This Question:
If "Which building are you currently located in?" "50 Milk Street" Is Selected
6. Which floor are you located on?

- ❍ Floor 5 (1)
- ❍ Floor 11 (2)
- ❍ Floor 12 (3)
- ❍ Floor 14 (4)
- ❍ Floor 15 (5)
- ❍ Floor 16 (6)
- ❍ Floor 17 (7)
- ❍ Floor 18 (8)

If "Which building (and floor) are you currently located in?" "1 Broadway" Is Selected
7. Which floor are you located on?

- ❍ Floor 3 (1)
- ❍ Floor 4 (2)
- ❍ Floor 5 (3)
- ❍ Floor 7 (4)
- ❍ Floor 9 (5)
- ❍ Floor 11 (6)
- ❍ Floor 14 (7)

If "Which building are you currently located in?" "101 Main Street" Is Selected
8. Which floor are you located on?

- ❍ Floor 1 (1)
- ❍ Floor 14 (2)
- ❍ Floor 15 (3)

Display the following question for clients in St. Louis
9. Which building are you currently located in?

- ❍ CET - Doris (1)
- ❍ CET - Lab (2)
- ❍ CIC@4240 (3)

***Questions have been removed here which asked about the client's experience at CIC, networking behaviors, and preferences about future locations.*

<u>Display the following questions for all clients</u>
Respondent Characteristics

10. What is your age?

- ❍ Under 25 (1)
- ❍ 25-34 (2)
- ❍ 35-44 (3)
- ❍ 45-54 (4)
- ❍ over 55 (5)
- ❍ Prefer not to say (6)

11. What is your gender?

- ❍ Male (1)
- ❍ Female (2)
- ❍ Non-binary / genderqueer (3)
- ❍ Prefer to self-describe: (4) ____________________
- ❍ Prefer not to say (5)

12. Were you born in the United States?

- ❍ Yes (1)
- ❍ No (2)
- ❍ Prefer not to say (3)

13. What is your race / ethnicity? Mark all that apply

- ❑ American Indian or Alaska Native (1)
- ❑ Asian (2)
- ❑ Black or African American (3)
- ❑ Hispanic/Latino/Spanish origin (4)
- ❑ Native Hawaiian or Pacific Islander (5)
- ❑ White (6)
- ❑ Other (7)
- ❑ Prefer to self-describe: (8) ____________________
- ❑ Prefer not to say (9)

14. Highest level of education

- ❍ No college education (1)
- ❍ Some college education (2)
- ❍ BA or equivalent degree (3)
- ❍ MA or equivalent degree (4)
- ❍ Doctorate or equivalent degree (5)

15. Field of highest degree:

- ❍ STEM field (1)
- ❍ Business or Economics (2)
- ❍ Other field (3)

16. Prior industry experience: Have you previously worked in the same industry as the current firm?
- ❍ Yes (1)
- ❍ No (2)

17. Prior start-up experience: Have you previously worked in a startup?
- ❍ Yes (1)
- ❍ No (2)

18. Prior start-up experience: Have you previously founded a business?
- ❍ Yes (1)
- ❍ No (2)

19. How many firms have you founded? (Previous or concurrent startups)
- ❍ 1 (1)
- ❍ 2 (2)
- ❍ 3 (3)
- ❍ 4 (4)
- ❍ 5 (5)
- ❍ 6 (6)
- ❍ 7 (7)
- ❍ 8 (8)
- ❍ 9 (9)
- ❍ 10 (10)
- ❍ >10 (11)

Display the following questions only for clients who have responded that they are Founder and/or CEO, Owner, or Other

Owner, Founder, or CEO Expectations

20. Facts about the current business: Number of employees
- ❍ 1 (1)
- ❍ 2 (2)
- ❍ 3 (3)
- ❍ 4 (4)
- ❍ 5 (5)
- ❍ 6-10 (6)
- ❍ 11-20 (7)
- ❍ 21-50 (8)
- ❍ More than 50 (9)

21. Your expectations regarding the future of this new firm. What would you expect the total sales, revenues, or fees to be in 5 years' time (from now)?
- ❍ Smaller than now (1)
- ❍ Same as now (2)
- ❍ Larger than now (3)
- ❍ More than 5 times larger than now (4)

22. In 5 years' time, how do you expect the company's employment to change in FTE?
- ❍ Smaller than now (1)
- ❍ Same as now (2)
- ❍ Larger than now (3)
- ❍ More than 5 times larger than now (4)

***Questions have been removed here which asked about sources of capital used to finance the firm.*

Financing
23. In total, how much external capital has been raised?
- ❍ Less than $250k (1)
- ❍ $250k - $900k (2)
- ❍ $900k - $3m (3)
- ❍ $3m - $9m (4)
- ❍ More than $9m (5)

Display the following questions for all clients
Innovation
24. Have you ever been an inventor on a patent?
- ❍ Yes (1)
- ❍ No (2)

25. Has your company worked on (or is currently working on) a new innovation?
- ❍ Yes (1)
- ❍ No (2)

Display This Question:
If "Has your company worked on (or is currently working on) a new innovation?" "Yes" Is Selected
26. Does your company patent these innovations?
- ❍ No (1)
- ❍ Yes, it already has some patents (2)
- ❍ Yes, it intends to patent in future (none yet) (3)

***Questions have been removed here which asked about the impact of other CIC clients on the firm.*

The next and final section uses personality assessment tools. It takes 3 minutes to complete.
Personality
27. How much do you typically enjoy taking risks?
(1= not at all happy to take risks; 10= very happy to take risks)
- 1 (not at all happy to take risks) (1)
- 2 (2)
- 3 (3)
- 4 (4)
- 5 (5)
- 6 (6)
- 7 (7)
- 8 (8)
- 9 (9)
- 10 (very happy to take risks) (10)

28. Some activities involve a "financial" risk, such as starting a business, investing, or gambling and betting — that is, there is a risk of losing money or other assets. In general, what is your propensity for accepting financial risks?
(1= very low; 10= very high)
- 1 (very low) (1)
- 2 (2)
- 3 (3)
- 4 (4)
- 5 (5)
- 6 (6)
- 7 (7)
- 8 (8)
- 9 (9)
- 10 (very high) (10)

How do the following statements agree with you? (1= strongly disagree; 5= strongly agree)

		1 Strongly Disagree	2	3	4	5 Strongly Agree
**29.**	I am talkative	O	O	O	O	O
**30.**	I am very thoughtful in my actions	O	O	O	O	O
**31.**	I am original, come up with new ideas	O	O	O	O	O
**32.**	I am reserved	O	O	O	O	O
**33.**	I am relaxed, handle stress well	O	O	O	O	O
**34.**	I have a forgiving nature	O	O	O	O	O
**35.**	I get nervous easily and worry	O	O	O	O	O
**36.**	I have an active imagination	O	O	O	O	O
**37.**	I am often lazy	O	O	O	O	O
**38.**	I value artistic, aesthetic experiences	O	O	O	O	O
**39.**	I am kind and considerate to others	O	O	O	O	O
**40.**	I do things efficiently	O	O	O	O	O
**41.**	I am social and outgoing	O	O	O	O	O
**42.**	If I work hard, I can successfully start a business	O	O	O	O	O
**43.**	Overall, my skills and abilities will help me start a business	O	O	O	O	O
**44.**	My past experience will be very valuable in starting a business	O	O	O	O	O
**45.**	I am confident I can put forth the effort needed to start a business	O	O	O	O	O
**46.**	I feel a great deal of pride when I complete a project successfully	O	O	O	O	O
**47.**	I have a strong desire to achieve positive results even when it requires a great deal of additional effort	O	O	O	O	O
**48.**	I surprise people with my novel ideas	O	O	O	O	O
**49.**	People ask me for help in creative activities	O	O	O	O	O
**50.**	I obtain more satisfaction from mastering a skill than coming up with a new idea	O	O	O	O	O
**51.**	I prefer work that requires original thinking	O	O	O	O	O
**52.**	I am not a very creative person	O	O	O	O	O

***Questions have been removed here related to business contacts and interest in future events. There was also an opportunity to provide any additional comments.*

53. All respondents can either choose to receive a $5 Amazon gift card, or to participate in a drawing for a $2,000 gift card of choice. We expect to receive around 1000 participants in the drawing. Your email will only be used for this purpose, and no additional questions or other contact attempts will be made using the email address provided below.
Please make your choice:

❍ Please send me a $5 Amazon gift card. My email is (1) ____________________

❍ Please enter me in a drawing for the $2,000 gift card. My email is (2) ____________________
